# Participation and the Well-Being of Older Adults with ADL Disabilities: A Longitudinal Application of the International Classification of Functioning, Health, and Disability

**DOI:** 10.3390/nursrep16070213

**Published:** 2026-06-25

**Authors:** Qiwei Li, Xiaoli Li, Cheng Yin

**Affiliations:** 1 Department of Public Health, California State University Fresno, Fresno, CA 93740, USA; 2 School of Health Sciences, Southern Illinois University, Carbondale, IL 62901, USA; xiaoli.li@siu.edu; 3 Department of Rehabilitation and Health Services, University of North Texas, Denton, TX 76201, USA; chengyin2@my.unt.edu

**Keywords:** well-being, activities of daily living, activity limitation, participation, NHATS, longitudinal, structural equation modeling, nursing

## Abstract

**Introduction**: Applying the International Classification of Functioning, Disability, and Health (ICF) framework, this study examined longitudinal associations among activities of daily living (ADL) limitations, participation, and well-being among community-dwelling older adults with ADL difficulty. **Methods**: We used five waves (2015–2019; Waves 5–9) of the National Health and Aging Trends Study (NHATS; baseline *n* = 5346). Well-being was measured using 11 NHATS items spanning affect, life satisfaction, and perceived control/self-efficacy. Participation was operationalized using five dichotomous indicators of engagement in common social and community activities. Autoregressive cross-lagged structural equation models were estimated using full-information maximum likelihood, and indirect associations were assessed with bootstrap standard errors. **Results**: We found that ADL limitations were associated with lower subsequent participation, while greater participation was associated with higher subsequent well-being across waves. Indirect associations linking ADL limitations to later well-being through participation were small and time-dependent. **Discussion**: Overall, the findings are consistent with an ICF-informed perspective in which participation is part of the longitudinal context linking activity limitations and well-being over time, although effect sizes were modest.

## 1. Introduction

As population aging accelerates worldwide, the number of older adults living with limitations in everyday functioning is projected to increase substantially in coming decades [1,2,3,4]. At the same time, policy and research attention has increasingly shifted toward aging in community settings, as most older adults prefer to remain in their own homes rather than institutional care [5,6,7,8]. Within this context, understanding how older adults with activity limitations maintain well-being while aging in place has become a central concern for gerontology and public health research [9,10].

Difficulty performing activities of daily living (ADLs), such as eating, bathing, dressing, toileting, or basic mobility, represents a common form of activity limitation in later life. ADL limitations reflect difficulty performing routine self-care and mobility activities rather than the presence of specific diseases, and they are among the most frequently reported forms of disability among older adults in the United States [1,11,12]. Because ADL limitations can constrain independence and daily activities, they are consistently associated with lower levels of participation and poorer well-being [13,14,15].

Traditional models of successful aging have often emphasized the avoidance of disease and disability, implicitly positioning older adults with functional impairments as aging “unsuccessfully” [16]. Although influential, this perspective has been critiqued for marginalizing a growing segment of the older population for whom aging with chronic conditions and activity limitations has become normative rather than exceptional [9,17]. These critiques have prompted calls for alternative frameworks that emphasize functioning, adaptation, and quality of life within social and environmental contexts rather than disease avoidance alone.

**Figure 1:**
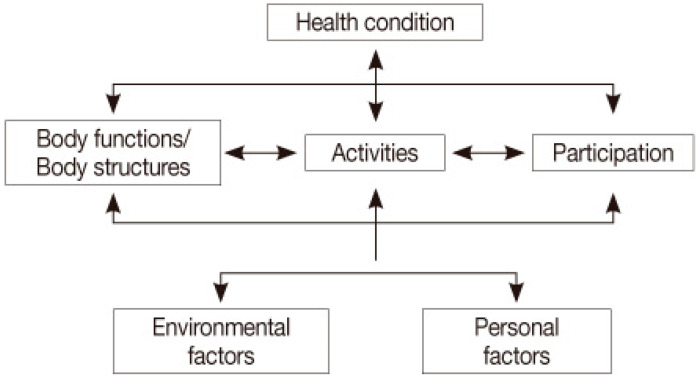
Interactions between the components of ICF [18].

One such framework is the World Health Organization’s International Classification of Functioning, Disability, and Health (ICF), which conceptualizes disability as a dynamic process shaped by interactions among body functions and structures, activities, participation, and contextual factors [18,19]. As demonstrated in Figure 1, within the ICF, activity limitations (such as ADL difficulty) and participation (involvement in life situations) are conceptually distinct but interrelated components of functioning [20,21]. This distinction has been widely adopted in gerontological research to examine how activity limitations translate into restrictions in social and community engagement [22,23].

Participation is a central construct within the ICF framework and refers to involvement in social, community, and civic life. In this study, the term community participation is used to describe observable indicators of the broader ICF construct of participation, operationalized using engagement in common social and community activities. Prior research has consistently shown that higher levels of participation are associated with better psychosocial outcomes among older adults, including greater life satisfaction and lower loneliness [24,25,26]. Among older adults with activity limitations, participation may provide opportunities to maintain social roles, autonomy, and a sense of meaning despite physical constraints [27,28,29].

Although well-being is not a discrete domain within the ICF taxonomy, it is commonly treated in ICF-informed aging research as a distal subjective outcome reflecting individuals’ evaluations of their lives and psychological functioning in context [30,31]. Subjective well-being encompasses emotional experiences, life evaluation, and perceived control or self-efficacy, all of which have been shown to be associated with participation and community engagement in later life [24,27]. From this perspective, participation may be prospectively associated with well-being by supporting social connectedness, role fulfillment, and adaptive coping, whereas activity limitations may indirectly undermine well-being by constraining opportunities for engagement.

Despite extensive cross-sectional evidence linking ADL limitations, participation, and well-being, longitudinal research examining how these constructs unfold over time remains limited, particularly among older adults with established activity limitations [15,32]. Much of the existing literature relies on cross-sectional designs that cannot assess temporal ordering or examine whether participation is prospectively associated with subsequent well-being after accounting for prior levels of functioning and psychosocial status.

Guided by an ICF-informed perspective emphasizing the distinction between activity limitation and participation, the present study uses five waves of data from the National Health and Aging Trends Study (NHATS) to examine longitudinal associations among ADL limitations, participation, and well-being among community-dwelling older adults with ADL difficulty. Specifically, this study assesses whether participation is prospectively associated with well-being over time and whether participation constitutes a statistical pathway linking ADL limitations and later well-being.

## 2. Methods

### 2.1. Data and Sample

Data were drawn from the National Health and Aging Trends Study (NHATS). NHATS is a nationally representative longitudinal study designed to examine late-life functioning, disability, and care among Medicare beneficiaries in the United States. Respondents are followed annually, and the survey collects detailed information on physical functioning, activity limitations, social participation, living arrangements, care, and subjective well-being.

The present study used data from Waves 5 through 9 (2015–2019). This analytic window was selected for two reasons. First, Wave 5 introduced a replenishment cohort, providing a refreshed nationally representative baseline for subsequent longitudinal analyses. Second, the five-wave period offers sufficient repeated observations for cross-lagged modeling while limiting complexity and instability associated with attrition in later waves.

Participants were included if they were aged 65 or older, resided in community settings, and reported at least one activity of daily living (ADL) limitation at baseline (Wave 5). The analytic sample sizes across waves were 5346 (2015), 4316 (2016), 3578 (2017), 3013 (2018), and 2588 (2019). Attrition over the study period was attributable to mortality, institutionalization, and study dropout, which is consistent with expected patterns in longitudinal aging research. The study was exempt from Institutional Review Board review because it used publicly available, deidentified data.

### 2.2. Measurements

#### 2.2.1. Subjective Well-Being

Well-being was assessed using 11 NHATS items commonly used to capture subjective well-being. These items reflect multiple components of subjective well-being, including emotional well-being (4 items), life satisfaction (4 items), and perceived control or self-efficacy (3 items). Together, these items capture individuals’ emotional experiences and evaluations of their lives, as well as psychological resources relevant to adaptation in later life.

Items assessing emotional well-being and life satisfaction were rated on 5-point frequency scales, whereas items assessing perceived control or self-efficacy were rated on 3-point agreement scales, consistent with NHATS survey design. Prior to scale construction, items were harmonized to ensure consistent directionality and comparability, and then averaged to create a composite well-being score, with higher values indicating greater subjective well-being. Internal consistency of the composite measure was acceptable (Cronbach’s α = 0.78).

#### 2.2.2. Participation

Participation was operationalized using five NHATS indicators reflecting engagement in common social and community activities: visiting friends or family in person, attending religious services, participating in clubs or organized activities, going out for enjoyment (e.g., dinner, movies, concerts), and engaging in volunteer work. Each activity was dichotomously coded (1 = *yes*, 0 = *no*), and responses were summed and averaged, with higher scores indicating participation in a broader range of activities.

NHATS includes follow-up items assessing whether health or functioning limited participation in some activities. Although these items are substantively relevant to the ICF framework, they were not incorporated into the current participation measure to maintain consistency across indicators and parsimony in longitudinal modeling. The implications of this measurement decision are addressed in the Limitations section.

#### 2.2.3. ADL Limitations (Activity Limitation)

ADL limitations were defined based on self-reported difficulty performing one or more basic self-care or mobility activities, including eating, bathing, toileting, dressing, getting out of bed, walking outside, and walking inside the home. Consistent with NHATS measurements, ADL limitation was defined based on reported difficulty with the activity and does not distinguish whether respondents used assistive devices or received help from others to complete the task.

A dichotomous indicator was used to reflect the presence of any ADL limitation (1 = *at least one ADL with reported difficulty*; 0 = *no reported difficulty*). This operationalization aligns with the study’s baseline inclusion criterion and facilitates comparability across waves, though it does not capture heterogeneity in how activities are completed. This limitation is discussed below.

### 2.3. Analytic Strategy

The analytic goal of this study was to examine longitudinal associations among ADL limitations, participation, and well-being over time within an ICF-informed framework. Because these constructs were expected to show temporal stability while also exhibiting cross-domain associations across repeated measurements, a longitudinal autoregressive structural equation modeling (SEM) approach was employed.

Autoregressive SEMs allow each construct to be predicted by its own prior value while simultaneously estimating cross-lagged associations among constructs across adjacent waves [33,34]. This approach permits assessment of whether ADL limitations at time *t* are associated with participation at time *t* + 1 and whether participation at time *t* is associated with well-being at time *t* + 1, after accounting for prior levels of each construct.

Model specification was guided by the ICF-informed distinction between activity limitation and participation. Accordingly, paths were specified from ADL limitations at time *t* to participation at time *t* + 1, and from participation at time *t* to well-being at time *t* + 1, along with autoregressive paths for each construct.

As an initial exploratory step, cross-sectional decomposition analyses were conducted separately for each wave using the paramed command in Stata to assess whether participation statistically accounted for the contemporaneous association between ADL limitations and well-being. These analyses were used as a diagnostic tool to evaluate whether the direction and magnitude of associations were consistent with the hypothesized longitudinal pathways, rather than to establish causal mediation.

In the longitudinal SEM, correlated residuals among ADL limitations, participation, and well-being within the same wave were included to account for shared variance attributable to unmeasured time-specific factors [35]. After estimating the theory-driven autoregressive cross-lagged model, modification indices and expected parameter change values were examined to identify localized areas of residual misfit. Three non-adjacent within-construct autoregressive paths were retained: ADL limitation from Wave 5 to Wave 7, participation from Wave 5 to Wave 7, and well-being from Wave 5 to Wave 7. These paths were theoretically interpretable as residual temporal dependence within the same construct that was not fully captured by adjacent-wave autoregressive paths. Because all three paths were limited to the same two-year interval from baseline to Wave 7 and were restricted to within-construct associations, this modification was applied conservatively rather than systematically across all waves or constructs. In longitudinal survey data, intermediate wave measures may contain measurement error or time-specific variation, such that adjacent-wave paths do not always fully absorb longer-term within-construct continuity. Importantly, these modifications did not introduce additional cross-domain pathways. Other potential non-adjacent autoregressive modifications were reviewed but not retained in order to preserve model parsimony.

Model fit was evaluated using multiple indices, including the chi-square test, RMSEA (<0.08), and comparative fit indices (CFI and TLI > 0.90). Information criteria (AIC and BIC) were used to compare nested models. Missing data were addressed using full-information maximum likelihood estimation. Indirect associations were estimated using bootstrapping with 500 replications to obtain standard errors for total, direct, and indirect effects. All analyses were conducted using Stata version 17 [36].

## 3. Results

### 3.1. Descriptive Statistics

**Figure 2:**
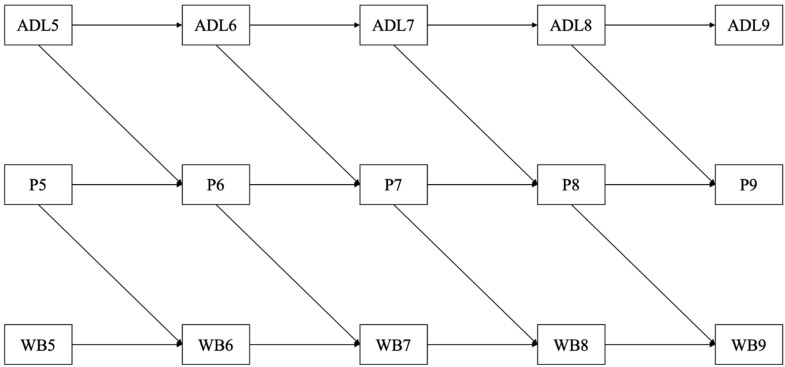
Conceptual autoregressive cross-lagged model linking ADL limitations, participation, and well-being. *Note.* Straight horizontal paths indicate within-construct autoregressive stability over time. Diagonal paths indicate hypothesized cross-lagged associations from ADL limitations to subsequent participation and from participation to subsequent well-being.

**Figure 3:**
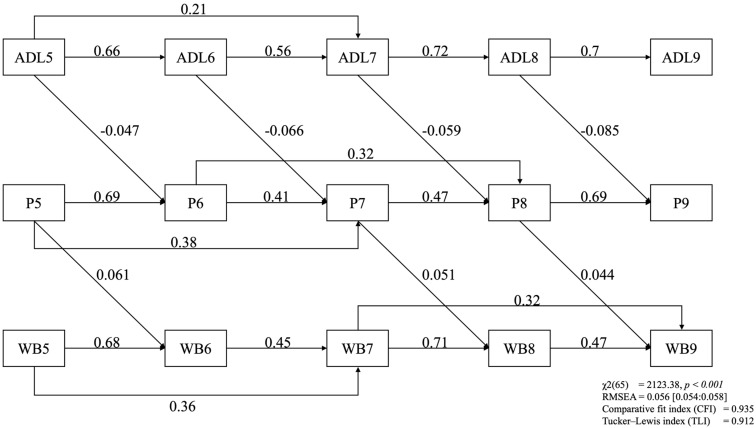
Final longitudinal SEM with standardized path coefficients. *Note.* The model includes autoregressive paths and hypothesized cross-lagged paths. Coefficients are standardized. Non-significant paths are omitted from the figure for readability.

The results are presented in three steps. First, Table 1 describes the demographic composition of the analytic sample at baseline and at the final follow-up wave. Second, Table 2 summarizes changes in ADL limitations, participation, and well-being across the five waves. Third, Figure 2 and Figure 3 and Table 3 present the longitudinal SEM results, including model specification, model fit, and indirect associations.

Table 1 presents baseline demographic characteristics of the analytic sample in 2015 (*n* = 5346) and 2019 (*n* = 2588). The age distribution shifted modestly over time, with a decrease in the proportion of participants aged 90 years and older (from 10.06% in 2015 to 6.53% in 2019). The proportion of female participants increased slightly from 58.45% to 61.55%. Racial composition remained relatively stable across waves, with White participants comprising the majority (64.61% in 2015; 65.73% in 2019), followed by Black (22.95% to 23.92%) and Hispanic participants (6.60% to 6.49%). Approximately 55% of participants were married at both time points.

Table 2 summarizes descriptive statistics for ADL limitations, participation, and well-being from 2015 through 2019. The proportion of respondents reporting at least one ADL limitation increased over time, from 0.17 (*SD* = 0.26) in 2015 to 0.31 (*SD* = 0.33) in 2019. Mean participation scores remained relatively stable across waves, ranging from 0.51 to 0.53. Mean well-being scores showed a gradual decline over time, decreasing from 6.55 (*SD* = 0.87) in 2015 to 6.34 (*SD* = 0.90) in 2019.

### 3.2. Structural Equation Models

Longitudinal structural equation modeling was used to examine temporal associations among ADL limitations, participation, and well-being. Consistent with prior recommendations for longitudinal modeling [33], correlations among repeated measures indicated substantial temporal stability across constructs. Correlations between ADL limitations measured at Wave 5 and Wave 9 ranged from 0.64 to 0.67; correlations for participation ranged from 0.70 to 0.72; and correlations for well-being ranged from 0.69 to 0.72.

A baseline autoregressive model was first estimated to assess stability of each construct over time. Standardized autoregressive coefficients indicated moderate to strong stability across adjacent waves for ADL limitations (0.66–0.73), participation (0.70–0.72), and well-being (0.71–0.73).

Cross-lagged paths were then added to examine associations between ADL limitations and subsequent participation, as well as between participation and subsequent well-being, consistent with the ICF-informed analytic framework (Figure 2). Across waves, reporting an ADL limitation was consistently associated with lower participation at the subsequent wave (β = −0.048 to −0.089, all *p* < 0.001). Participation was positively associated with subsequent well-being across waves (β = 0.033 to 0.081, all *p* < 0.001).

To account for shared variance attributable to unmeasured time-specific factors, correlated residuals among ADL limitations, participation, and well-being within the same wave were included. The inclusion of these correlations improved overall model fit while leaving the magnitude and direction of the primary cross-lagged associations largely unchanged.

The final model incorporated covariates and selected wave-skipping autoregressive paths to capture longer-term stability within constructs. Model fit indices indicated good fit to the data (CFI = 0.935; TLI = 0.912; RMSEA = 0.056, 95% CI [0.054, 0.058]). Standardized autoregressive coefficients remained moderate across constructs, and cross-lagged associations between ADL limitations and participation, as well as between participation and well-being, were retained (ADL → participation β = −0.047 to −0.085; participation → well-being β = 0.014 to 0.061).

### 3.3. Indirect Associations

Indirect associations linking earlier ADL limitations to later well-being through participation were examined across three temporal sequences: Wave 5 → Wave 7, Wave 6 → Wave 8, and Wave 7 → Wave 9. One indirect path (participation at Wave 6 to well-being at Wave 7) was not statistically significant and was excluded from the final model to maintain parsimony.

Time-specific indirect associations varied in magnitude and statistical significance. The indirect association from ADL limitations at Wave 5 to well-being at Wave 7 through participation was small and not statistically significant (β = −0.001, SE = 0.002, *p* = 0.273). Indirect associations from Wave 6 to Wave 8 (β = −0.004, SE = 0.004, *p* = 0.001) and from Wave 7 to Wave 9 (β = −0.003, SE = 0.004, *p* = 0.017) were statistically significant but modest in magnitude. The cumulative indirect association from ADL limitations at Wave 5 to well-being at Wave 9 through participation was −0.005 (SE = 0.005, *p* < 0.001). The corresponding total association between ADL limitations at Wave 5 and well-being at Wave 9 was not statistically significant (*p* = 0.237), indicating that the indirect pathway accounted for a modest portion of an overall weak long-term association.

For comparison, cross-sectional decomposition analyses conducted at each wave indicated that participation statistically accounted for approximately 16.0% to 21.4% of the contemporaneous association between ADL limitations and well-being (Table 3; Figure 3). These results are descriptive and are presented to contextualize the longitudinal findings rather than to support causal mediation.

## 4. Discussion

This study examined longitudinal associations among ADL limitations, participation, and well-being among community-dwelling older adults with ADL difficulty using a nationally representative, multi-wave dataset. Guided by an ICF-informed perspective, the analyses focused on temporal patterns linking activity limitation, participation, and well-being rather than on causal inference. Overall, the findings indicate that ADL limitations, participation, and well-being exhibit substantial temporal stability, while also showing consistent associations across adjacent waves. The findings should therefore be interpreted as evidence of longitudinal associations among empirically measured indicators of activity limitation, community participation, and subjective well-being, rather than as a comprehensive test of all components of the ICF framework.

Across the five-year period, the proportion of older adults reporting ADL difficulty increased modestly, participation levels remained relatively stable, and well-being declined slightly. Each construct was strongly predicted by its prior value, highlighting the importance of historical status in shaping subsequent functioning and well-being in later life. These patterns are consistent with prior longitudinal research demonstrating that functional status, social engagement, and subjective well-being tend to show persistence over time among older adults.

Consistent with previous studies, ADL limitations were negatively associated with well-being, and participation was positively associated with well-being among older adults with activity limitations [15,37]. Extending prior work, the present study examined these associations longitudinally and found that participation was prospectively associated with subsequent well-being across multiple waves, even after accounting for prior participation and well-being. Although the magnitude of these associations was modest, their consistency across waves suggests that participation remains relevant for understanding well-being among older adults with ADL difficulty.

The analyses also indicated that participation statistically accounted for a portion of the longitudinal association between ADL limitations and well-being. Indirect associations linking earlier ADL limitations to later well-being through participation varied across waves, with some pathways reaching statistical significance and others not. These findings suggest that participation may play a partial and time-dependent role in linking activity limitations and well-being rather than serving as a uniform explanatory mechanism. This pattern aligns with the complexity of aging processes in community settings, where well-being is shaped by multiple functional, social, environmental, and psychological factors.

From a theoretical perspective, the observed temporal patterns are broadly consistent with the ICF framework, which emphasizes interactions among activity limitation, participation, and broader indicators of health and functioning within specific environmental contexts. Rather than treating disability as a static outcome, the findings illustrate how activity limitations and participation are dynamically related over time among community-dwelling older adults. While the results do not establish causal pathways, they provide empirical support for applying an ICF-informed perspective to longitudinal research on aging and well-being. At the same time, the empirical measures used in this study represent narrower operational indicators than the full ICF framework. Thus, the findings support the usefulness of the ICF distinction between activity limitation and participation, while also demonstrating the need for more refined measurement of these constructs in future longitudinal research.

### 4.1. Implications

Well-being is a central component of quality of life in later adulthood, particularly for older adults aging in place with activity limitations. The present findings highlight substantial within-construct stability and consistent cross-domain longitudinal associations among ADL limitations, participation, and well-being, suggesting that both activity limitation and social engagement are relevant for understanding well-being trajectories among community-dwelling older adults.

Although ADL limitations were relatively stable over time, they were consistently associated with lower subsequent participation. This pattern suggests that activity limitations may constrain opportunities for engagement in social and community activities. Efforts aimed at supporting mobility, accessibility, or adaptive functioning may therefore be relevant for maintaining participation among older adults with ADL difficulty, though such implications should be interpreted cautiously given the observational nature of the data.

Participation levels remained relatively consistent across waves and were associated with subsequent well-being. These findings suggest that participation may be an important correlate of well-being among older adults with ADL limitations. Community-based approaches that facilitate social contact, transportation, or accessible activities—such as senior centers or community programs—may help support engagement among older adults with activity limitations. Importantly, these implications are suggestive rather than prescriptive and warrant further evaluation using intervention or quasi-experimental designs.

The findings also have implications for future research using the ICF framework. Future studies should distinguish more explicitly between activity limitation, observable participation, perceived participation restriction, and subjective well-being. In addition, longitudinal research should examine whether the association between participation and well-being differs by severity of ADL limitation, type of activity limitation, gender, living arrangement, caregiving support, and environmental context. Such work would help clarify when and for whom participation is most strongly associated with well-being.

### 4.2. Limitations

Several limitations should be acknowledged. Participation was measured using a limited set of dichotomous indicators capturing observable engagement in common social and community activities rather than the full conceptual scope of participation as defined in the ICF, and follow-up items assessing participation restriction due to health or functioning were not incorporated. As a result, the participation measure did not capture frequency of engagement, perceived importance of activities, satisfaction with participation, quality of social interaction, or environmental barriers to participation. Although this decision reduced conceptual overlap between ADL limitation and participation restriction, it also means that the present measure captured observable activity engagement rather than the broader ICF construct of participation. Future studies should examine whether richer measures of participation restriction, environmental accessibility, and perceived participation meaning produce different or more nuanced longitudinal associations with well-being.

ADL limitation was operationalized as a binary indicator of any reported difficulty, which does not distinguish between individuals who complete activities independently, use assistive devices, or rely on help from others, despite NHATS providing richer information on activity performance. This binary operationalization also does not distinguish mild from severe limitations or capture differences across specific ADL domains. Therefore, the findings should be interpreted as applying to the presence of any ADL difficulty rather than to the severity, duration, or type of functional limitation.

Although longitudinal data and autoregressive modeling were used, the analyses remain observational and focus on temporal associations rather than causal inference, and the models specified unidirectional cross-lagged paths without incorporating potential reciprocal feedback processes among activity limitation, participation, and well-being that are explicitly acknowledged within the ICF framework. In addition, well-being was measured as a composite construct encompassing emotional well-being, life satisfaction, and perceived control or self-efficacy; although this approach demonstrated acceptable internal consistency and aligns with prior NHATS-based research, these components represent related but distinct psychological domains. Finally, attrition and selective survival may have influenced observed associations despite the use of full-information maximum likelihood estimation. Because older adults with greater disability or poorer health may be more likely to die, enter institutional settings, or be lost to follow-up, the later-wave analytic sample may underrepresent those with the most severe limitations. Additionally, NHATS data collection tools were designed primarily for U.S. community-dwelling older adults, which should be considered when interpreting these findings or applying them to other populations.

## 5. Conclusions

Using five waves of nationally representative NHATS data, this study examined longitudinal associations among ADL limitations, participation, and subjective well-being among community-dwelling older adults with ADL difficulty. The findings indicate that participation is prospectively associated with subsequent well-being over time and constitutes a modest, time-dependent statistical pathway linking activity limitation and later well-being. Although effect sizes were small and the analyses do not support causal inference, the results are consistent with an ICF-informed perspective emphasizing the distinction between activity limitation and participation. Together, these findings suggest that participation warrants continued consideration as part of the broader context in which older adults with functional limitations experience and maintain well-being while aging in place, and they highlight the value of longitudinal approaches for understanding dynamic processes in later life.

## Figures and Tables

**Table 1:** Demographic characteristics of the analytic sample at baseline and final follow-up wave.

	2015 (*n* = 5346)		2019 (*n* = 2588)	
Age	*n*	%	*n*	%
65–69	679	12.7	322	12.44
70–74	1146	21.44	622	24.03
75–79	1115	20.86	585	22.6
80–84	1064	19.9	535	20.67
85–89	804	15.04	355	13.72
90+	538	10.06	169	6.53
Gender				
Male	2221	41.55	995	38.45
Female	3125	58.45	1593	61.55
Race				
White	3454	64.61	1701	65.73
Black	1227	22.95	619	23.92
Hispanic	353	6.6	168	6.49
Other	312	5.84	100	3.86
Marital status				
Married	2946	55.11	1421	54.91
Not married	2400	44.89	1167	45.09

*Note.* The 2015 column describes the baseline analytic sample. The 2019 column describes respondents from the analytic sample with available follow-up data in Wave 9. The reduction in sample size across waves reflects expected longitudinal attrition in NHATS, including mortality, nonresponse, and loss to follow-up. Percentages are calculated within each wave-specific analytic sample.

**Table 2:** Descriptive Statistics of ADL, Participation, and Well-being From 2015 to 2019.

	2015	2016	2017	2018	2019
	M (*SD*)	M (*SD*)	M (*SD*)	M (*SD*)	M (*SD)*
ADL	0.17 (0.26)	0.22 (0.30)	0.24 (0.31)	0.27 (0.32)	0.31 (0.33)
Participation *	0.53 (0.27)	0.53 (0.27)	0.52 (0.27)	0.51 (0.27)	0.51 (0.27)
Well-being	6.55 (0.87)	6.46 (0.89)	6.43 (0.89)	6.37 (0.90)	6.34 (0.90)

*Note.* * Participation refers to the study variable measuring engagement in social and community activities, not survey participation.

**Table 3:** Iterations of SEM and Model Fits.

	*χ* ^2^	CFI	TLI	RMSEA
M1: Theoretical model	4252.72	0.862	0.829	0.098
M2: Added within-wave residual covariances	3804.33	0.880	0.839	0.094
M3: Added covariates	3409.74	0.890	0.844	0.074
M4: Added covariates and selected non-adjacent autoregressive paths	2122.18	0.935	0.911	0.056

## Data Availability

The data presented in this study are openly available in National Health and Aging Trends Study. Produced and distributed by www.nhats.org with funding from the National Institute on Aging and Office of Behavioral and Social Sciences Research (OBSSR) (grant number U01AG032947).

## Public Involvement Statement

No public involvement in any aspect of this research.

## Guidelines and Standards Statement

This manuscript was drafted against the Strengthening the Reporting of Observational Studies in Epidemiology (STROBE; [38]) Statement for observational research.
